# Benzoyl Valine Quasiracemates: Pairing CF_3_ Quasienantiomers with H to *t*-Butyl

**DOI:** 10.1021/acs.cgd.4c00307

**Published:** 2024-04-15

**Authors:** Ashah M. Gould, Danielle R. Schalk, Molly E. Fleagle, Kraig A. Wheeler

**Affiliations:** Department of Chemistry, Whitworth University, 300 West Hawthorne Road, Spokane, Washington 99251, United States

## Abstract

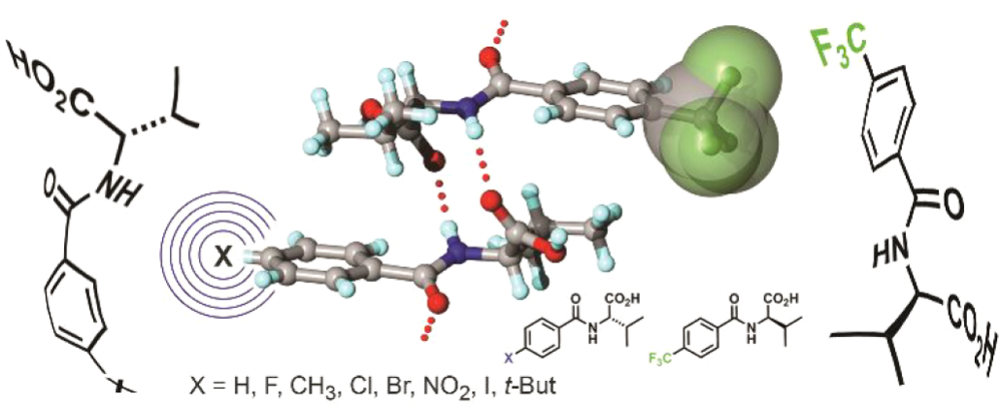

Understanding the interplay of structural features responsible for molecular assembly is essential for molecular crystal engineering. When assembling molecules with encoded motifs, *first choice* supramolecular strategies almost always include robust directional nonbonded contacts. Quasiracemic materials, considered near racemates since cocrystallization occurs with chemically unique components, lack a molecular framework or functional group restrictions, highlighting the importance of molecular shape to molecular assembly. Recently, our group reported quasiracemates derived from benzoyl leucine/phenylalanine derivatives with two points of chemical difference. In this study, we modified the chemical framework with valine and increased the scope of the work by imposing a larger variance in the side chain substituents. Pairing a CF_3_ component with quasienantiomers that differ iteratively from hydrogen to *t*-butyl offers an important view into the supramolecular landscape of these materials. Single-crystal X-ray crystallography and lattice energy assessments, coupled with conformational and crystal structure similarity searches, show an elevated degree of isomorphism for many of the targeted 17 racemates and quasiracemates. These benzoyl amino acid molecular architectures create extended hydrogen-bond patterns in the crystal that provide enhanced opportunities to study the shape space and molecular recognition profiles for a diverse family of quasienantiomeric components.

## Abstract 2

A family of benzoyl valine racemates and quasiracemates cocrystallize with a high degree of isomorphism despite significant differences in the shape space of the quasienantiomeric components.

## Introduction

The legacy of Dr. Olga Kennard to the science community spans more than half a century, with focused efforts in the early 1960s developing the Cambridge Structural Database^1^ (CSD) under the newly established Cambridge Crystallographic Data Centre (CCDC). The CSD started from an archive of a few thousand structures that today celebrates a collection of nearly 1.3 M unique curated entries from 500,000 authors. Many structurally rich disciplines in the Materials and Life Sciences can trace their growth partly to the CSD, the creation of CCDC Mercury^2^ in the 2000s, and the full suite of cheminformatics software currently offered by the CCDC. The influence of the CSD on emergent and established chemical models and principles should not be understated. Crystallographic data has contributed to our understanding of several important aspects of chemical dynamics and molecular contacts (metal–ligand, guest–host and nonbonded) that were once disputed and thought unlikely, if not impractical.^3−15^ The potency of a well-placed crystal structure or collections of crystallographic data continues to offer unique clarity to relevant science topics and applications that are largely intractable by using other material structure methods.

**Scheme 1:**
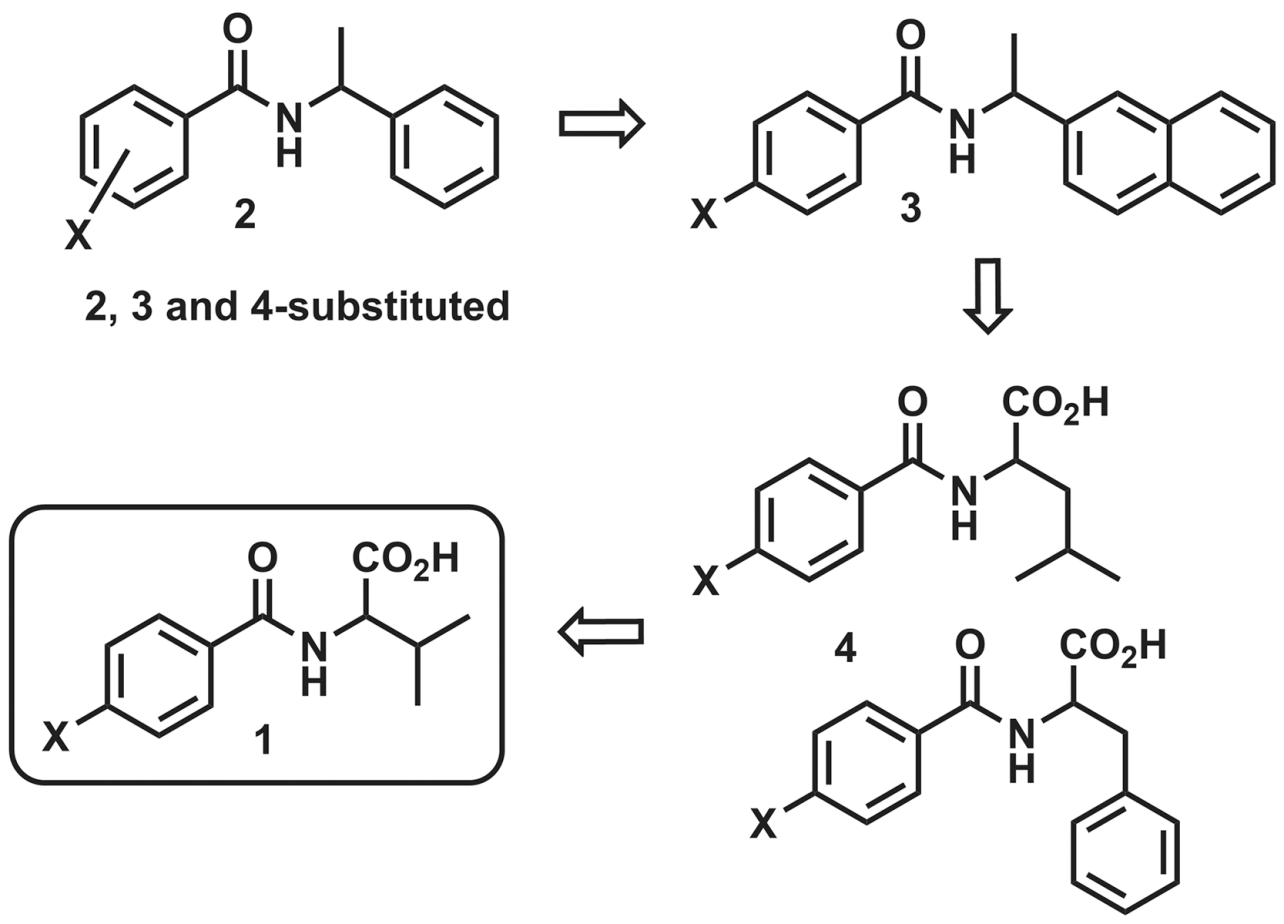
Benzoyl valine (**1**) chemical framework targeted by this study. Other recently investigated molecular frameworks, diarylamide (**2**), naphthylamide (**3**), and benzoyl leucine phenylalanine (**4**), utilized in quasiracemic studies.

This special *Crystal Growth & Design* issue, dedicated to Dr. Kennard, offers a testament to the significance of her contributions to the science community. It is important to note that the work presented here directly benefits from many CCDC resources Dr. Kennard established and championed. Several tangible benefits to this study include internal databases, conformational comparisons targeted in the CSD database, and crystal packing similarity assessments. Additionally, the molecular interactions observed in our systems build on previous CSD-supported investigations that helped to codify the details of nonbonded contacts and their conditional exceptions. Here, we highlight our effort to understand the molecular recognition profiles of small-molecule cocrystalline assemblies and how the design elements of molecular shape and hydrogen bonds can be used to advantage for creating predictable near-inversion-related molecular assemblies. This work probes the shape space of quasiracemic materials^16,17^ and their ability to assemble pairs of topologically comparable but chemically unique quasienantiomeric components constructed from benzoyl valine (**1**) molecular frameworks (Scheme 1). Similar to recent reports from our group describing the quasiracemic behavior of 2, 3 and 4-substituted diarylamide^18,19^ (**2**), 4-substituted naphthylamide^20^ (**3**) and 4-substituted benzoyl leucine/phenylalanine^21^ (**4**) systems, the framework based on **1** consists of chiral components (valine) and easily derivatized benzoyl components in the 4-position that, together, promote the construction of homologous sets of quasienantiomeric building blocks. Progress from using molecular architectures based on **2** and **3** to benzoyl valine **1** offers a fundamental change in the quasiracemate design approach, where increased crystal lattice stabilization is achieved by creating more extensive hydrogen-bond networks *via* an added carboxyl group. As seen with the previous benzoyl leucine/phenylalanine quasiracemates (**4**), we anticipate that quasiracemates based on benzoyl valine **1** should also provide opportunities to cocrystallize quasienantiomeric components. By pairing homochiral **1**-CF_3_ with a set of eight spatially engineered quasienantiomers (X = H, F, CH_3_, Cl, Br, NO_2_, I, and *t*-Bu), this study seeks to understand the structural boundary of molecular recognition when the CF_3_ antipode encounters other quasienantiomers of varying size and shape. The interest in the CF_3_ substituent as the hinge point in these quasiracemate studies stems from its underrepresented use in known quasiracemates, comparative size, and the potential impact of the group’s significant electronegativity on quasiracemic assemblies. The series of related H to *t*-Bu racemic compounds is also included in this study to examine the structural mimicry of the quasiracemic structures of these compounds.

## Experimental Section

### Synthetic Procedure

All chemicals and solvents were purchased from MilliporeSigma and VWR Scientific and used as received without further purification unless stated otherwise. The synthetic strategy used to prepare the 4-substituted benzoyl valine derivatives was adapted from previously reported methods.^22,23^ The following general procedure, as described for *N*-benzoyl-l-valine (l-**1**-H), was used to generate the homologous series of benzoyl valine **1** compounds.

l-Valine (0.185 g, 1.58 mmol) and 1.55 mL of 1 M NaOH (1.55 mmol) were added to a 25 mL round-bottom flask and stirred at room temperature for 5–10 min until dissolution. Benzoyl chloride (0.206 g, 1.46 mmol) and 1.55 mL of 1 M NaOH (1.55 mmol) were added simultaneously at 0 °C to the clear colorless mixture over 5 min. The reaction was warmed to room temperature and stirred for an additional 2 h. The reaction was then cooled to 0 °C, acidified with 6 M HCl to a pH of 2–3, and filtered to give l-**1**-H as a colorless solid (0.213 g, 0.964 mmol, 66% yield). Reaction yields of 52–95% were attained for the family of chiral benzoyl valine derivatives. See the Supporting Information for NMR assignments for **1**.

### Crystal Growth and Single Crystal X-ray Diffraction

The racemic and quasiracemic cocrystals prepared for this study used slow evaporation of methanolic solutions with equimolar mixtures (10–20 mg scale) of the l and d forms of the components. Crystals suitable for X-ray diffraction studies were retrieved after 1–5 days.

The diffraction data on single crystals were collected at 100 Κ by using a Bruker D8 Venture IμS microfocus dual-source diffractometer equipped with a PHOTON II CPAD detector and an Oxford cryogenic system. Monochromatic Cu Kα (λ = 1.54178 Å) or Mo Kα (λ = 0.71073 Å) radiation was used in the data collection using phi (φ) and omega (ω) scan strategies. Cell measurement, data collection, integration, scaling, and absorption correction were achieved using the APEX4 software.^24^ Data were processed using SAINT,^24^ and an absorption correction was performed using SADABS^24^ programs incorporated in APEX4. The structure was solved using SHELXT^25^ and refined using the SHELXL^26^ program, both implemented in OLEX2.^27^ The non-hydrogen atoms were located in successive difference Fourier syntheses and refined with anisotropic thermal parameters. The CH hydrogen atoms were placed at calculated positions and refined using a riding model with appropriate HFIX commands and U_iso_ = 1.2 × U_eqiv_ (1.5 × U_eqiv_ for methyl groups). NH and OH hydrogen atoms were located in difference Fourier syntheses (NH, 1.2 × U_eqiv_; OH, 1.5 × U_eqiv_), and their positions were refined independently. The N–H and O–H distances were restrained to 0.85(2) Å using a DFIX command when appropriate. The program OLEX2^27^ was used for packing analysis and molecular graphics. Crystallographic data and hydrogen-bond parameters are summarized in Tables S1 and S2, respectively.

### Crystal Lattice Energy Determinations

Crystal lattice energies (*E*_Latt_) were determined for the 17 crystal structures included in this study by analyzing residue-to-residue contacts. The software package Crystal Explorer,^28^ equipped with Gaussian16,^29^ was used to create molecular assemblies starting from a cluster radius of 10 Å with *E*_Latt_ values computed by direct summation of interaction energies (i.e., electrostatic, dispersion, polarization, and repulsion) using the central molecule and molecules included in the cluster. Several benzoyl valine **1** structures [(±)-**1**-CF_3_, d-**1**-*t*-Bu/l-**1**-CF_3_] exhibited disorder of the CF_3_ group. Lattice calculations were performed on each disorder component separately, and their contribution was weighted by using the occupancy factors established from the crystallographic data. Final *E*_Latt_ values for the *Z’* > 1 structures [(±)-**1**-H, (±)-**1**-F, [(±)-**1**-CH_3_, l-**1**-H/d-**1**-CF_3_] were determined by averaging the contributions from each symmetry-independent molecule.

### CCDC Cambridge Structural Database Search

The CSD^1^ (vs 5.43) searches were restricted to organic structures with 3D coordinates. A search of valine (52), valinate (47), and valinyl (99) fragments in the database resulted in 228 hits. Duplicate structures retrieved from the valine and valinate searches were removed from the valinyl data set. If necessary, the structures were inverted to the *S* configuration and the N–C_β_–C_γ_–H (ϕ_1_) and N–C_β_–C_α_–O (ϕ_2_) dihedral angles were tabulated. Two N–C_β_–C_α_–O angles were retrieved for each valinate and valine entry, with the larger value retained and used in the conformational analysis.

## Results and Discussion

### Molecular Shape Descriptors

Because quasiracemic materials require only pairs of chemically unique molecules of opposite handedness, it is unsurprising that the collection of quasiracemates reported in the literature lacks the constraints of chemical class, molecular framework, and functional group selections. This flexibility in the design strategy of quasiracemates also holds for nonbonded contacts, where applying specific intermolecular interactions to quasiracemic assemblies can offer tangible benefits to the molecular assembly processes but is largely unnecessary when constructing these cocrystalline systems. While many factors contribute to the organization of quasiracemic materials, one mutually shared structural feature that pervades all known quasiracemates is the complementary shapes of the quasienantiomeric components. Analogous to enantiomer topologies and their effect on racemate cocrystallization, the shape space of quasienantiomers provides a primary driving force for molecular alignment due to the close packing of the components. One indication of the importance of molecular shape to the construction of quasiracemic assemblies is that the efficient alignment realized from assembling quasienantiomers (and enantiomers) can not be achieved in single-component homochiral systems. Additionally, this molecular shape dependence from quasiracemic assemblies is evident from inspection of the small-molecule organic crystal structures, where >90% prefer to crystallize in centrosymmetric space groups.^30−32^ Quasiracemates, with only a few exceptions, cocrystallize in analogous motifs best described by near-inversion symmetry alignment, because of the chemically distinct quasienantiomeric components.

Given the importance of molecular topology to quasiracemate formation, several persistent issues that relate to the molecular shape features of quasiracemic materials exist. Several persistent questions include: (i) can the difference in the molecular shape of quasienantiomers be quantified and (ii) how closely do quasiracemic assemblies mimic the true symmetry observed in the crystal structures of the racemic counterparts? Developing these areas will require methods that effectively manage the structural challenges confronted by many quasiracemic components and adequately describe the shape space of molecules. Several geometric approaches and computer-aided discovery models show promise for profiling the properties of molecular shape but lack direct applicability to quasiracemate materials.^33−38^ Similar challenges with current crystallography applications exist, such as the molecular overlay feature in CCDC Mercury^2^ and the near-symmetry estimates in Avnir’s Continuous Symmetry Measures,^39,40^ where atom pairs are required for these assessments. This difficulty stems from quasiracemic systems constructed from components differing in the number of atoms or atom types. Since processing the crystallographic data requires removing the atoms responsible for the molecular difference, alternative methods are needed that allow for the variation in quasiracemic components. Our recent efforts in this area showed the value of using an in-silico method based on a 3D grid approach.^19^ The development of this diagnostic tool for comparing the shape space of pairs of quasienantiomers is ongoing and will be the focus of a future report.

**Figure 1:**
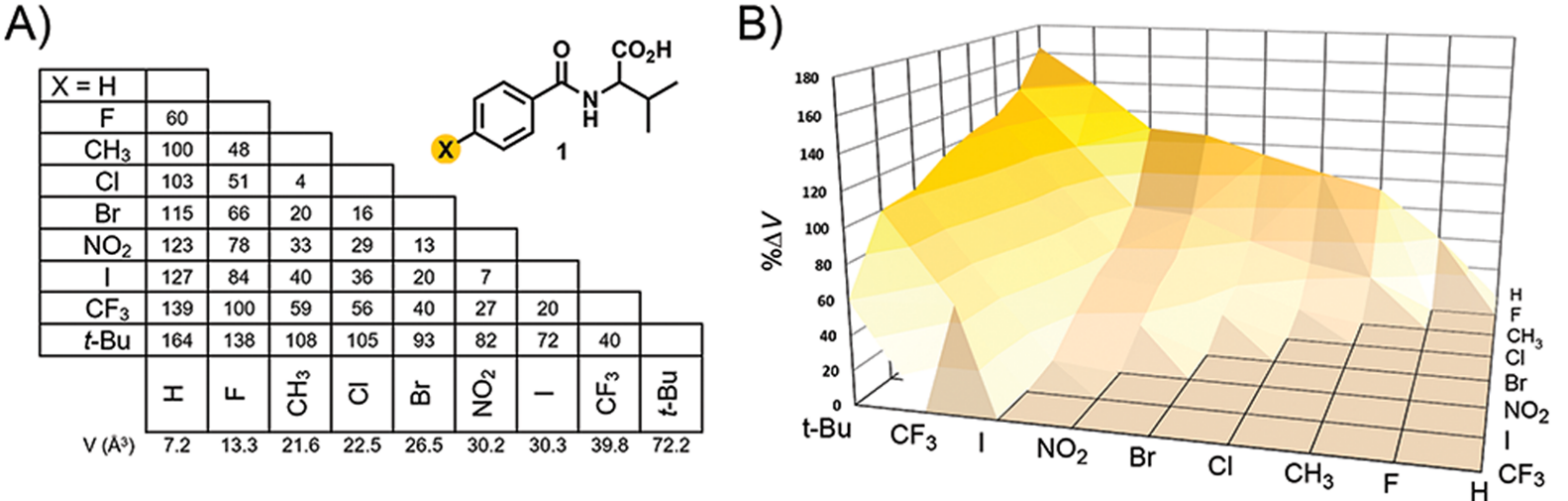
Benzoyl valine **1** showing percent volume differences (%Δ*V*) of functional groups X depicted in (A) table and (B) topological map formats.

Without adequate topological descriptors, our group^21^ and others^41,42^ have extensively used the property of volume to compare molecules and functional groups. The volumes for substituents X were compiled from previous reports.^19,41^ While evaluating volumes provides a quantitative and easy tool for quasienantiomer comparison, this structural parameter does not directly represent the shape space of substituents or molecules. For example, the design strategy of this study includes probing the structural boundary of molecular assembly by pairing quasienantiomeric components with pendant substituents ranging from H to *t*-butyl. The volumes of these groups and the percent difference (%Δ*V*) are provided in Figure 1 (%Δ*V* = [(|*V*_1_ – *V*_2_|/(*V*_1_ + *V*_2_)/2] × 100)). The drawback with the volume approach is apparent when considering specific functional group pairs. For Br and NO_2_, the group volumes are nearly equivalent (26.5 and 30.2 Å^3^, respectively), though their shapes are quite distinct (i.e., spherical vs trigonal planar). While the current effort continues to rely on volume-based assessments to explain quasiracemate behavior, it also recognizes the need for additional tools that would provide benefit to the structural community. The suite of resources developed by the CCDC in the last few decades continues to equip researchers in ways not previously envisioned, even in the recent past. Moving forward with efforts to define the complete landscape of molecular features responsible for molecular assembly demands a concerted effort from the entire structural community, where the likes of the CCDC and other enterprises, including academia, are needed to untangle solutions to these growing needs.

### Crystallographic Assessments

Crystal structure determinations of the benzoyl valine **1** compounds were undertaken to understand the crystal organization preferences for this family of racemic and quasiracemic materials. Because the design strategy includes pairing **1**-CF_3_ with sets of quasienantiomers, where X = H, F, CH_3_, Cl, Br, NO_2_, I, and *t*-Bu (Figure 1), it is expected that the systematic use of molecular shape to molecular assembly will provide critical insight into the molecular recognition profiles of these materials. Our recent experience of cocrystallizing diarylamide^18,19^ (**2**) and naphthylamide^20^ (**3**) quasiracemates proved more challenging compared to systems constructed from the benzoyl leucine and phenylalanine^21^ (**4**) architectures. This observation follows an increase in the number of hydrogen-bond donor and acceptor atoms available with **4**. For amides **2** and **3**, the solitary N–H···O=C hydrogen bonds create C(4) graph set patterns,^43,44^ whereas systems of **4** incorporating amino acid components generate extensive networks of *R*_2_^2^(10) and C(7) or *R*_2_^2^(8) and C(4) N–H···O and O–H···O interactions.^21^ Similar to the effect of a larger chemical framework on crystal lattice stabilization (*e.g.,***2** to **3**) we also anticipated additional hydrogen-bond acceptor and donor atoms would translate to improved crystal stabilization.

Our previous work in this area supports the notion that thermodynamically more stable crystalline phases create new opportunities to explore quasiracemates constructed from a greater spatial diversity. When considering the case of the aryl amide systems, success was achieved with the diarylamide **2** quasiracemates when using components of comparable size and shape (%Δ*V* < 60%) [2-substituted: H/F, Cl/Br, CH_3_/CF_3_, NO_2_/Br, NO_2_/CF_3_, and CF_3_/I; 3-substituted: H/F, CH_3_/Cl, Br/CF_3_; and 4-substituted: H/F, CH_3_/NO_2_, and OCH_3_/Br]. Employing the spatially larger naphthylamide **3** frameworks produced an H/NO_2_ quasiracemate with %Δ*V* = 123%. More recently, focus on the benzoyl leucine and phenylalanine **4** systems permitted quasiracemates with two points of structural difference by pairing quasienantiomeric components differing by H/CF_3_ (%Δ*V* = 139%) and Leu/Phe (%Δ*V* = 18%). This study builds on these previous results and expands our understanding of the molecular recognition properties of quasiracemates using benzoyl valine **1**, by combining the quasienantiomer **1**-CF_3_ with nine homochiral components that differ incrementally in size and spatial properties.

**Figure 2:**
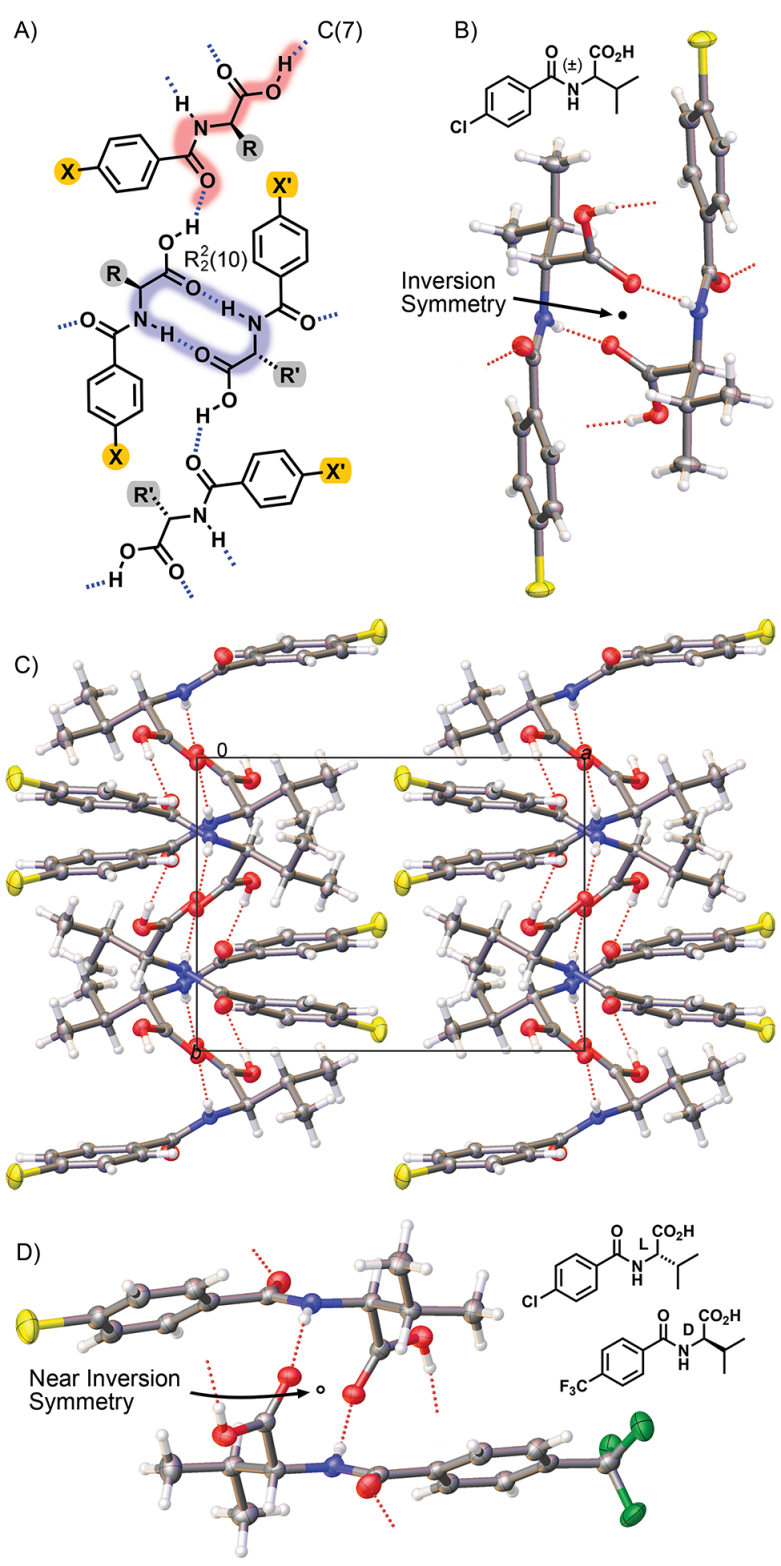
Projections showing Form I (A) hydrogen-bond motifs, (B,C) crystal structure views of (±)-**1**-Cl, and (D) the crystal structure of quasiracemate l-**1**-Cl/d-**1**-CF_3_.

A total of 17 quasiracemic (eight) and racemic (nine) crystal structures were included in this investigation. Because the crystal structures of quasiracemates frequently imitate the crystal packing observed in the component racemates, this structural information is often helpful in understanding the observed structural trends. All investigated racemic and quasiracemic systems exhibit *R*_2_^2^(10) and C(7) hydrogen-bond motifs (Figure 2A). The N–H···O_carboxyl_ driven cyclic *R*_2_^2^(10) graph set patterns offer a noteworthy departure from the frequently encountered *R*_2_^2^(8) motifs^45^ found in crystal structures of carboxylic acids. Most of the 17 crystal structures display a striking similarity and are assigned to Form I. These isomorphic structures organize in space groups *P*2_1_ (quasiracemates) or *P*2_1_/*c* (racemates), with similar unit cell parameters and crystal packing motifs. The racemates with X = Cl, Br, NO_2_, CF_3_, I, and *t*-Bu and quasiracemates constructed from **1**-CF_3_ and quasienantiomers (X = F, CH_3_, Cl, Br, NO_2_, I, and *t*-Bu) organize in the crystal using Form I (13 structures). Figure 2B–D shows Form I crystal packing using the (±)-**1**-Cl and l-**1**-Cl/d-**1**-CF_3_ structures as examples. The components align to give N–H···O=C *R*_2_^2^(10) patterns that reside on inversion (racemates) or near-inversion (quasiracemates) symmetry elements. These interactions create 2D hydrogen-bond patterns propagating in the *bc* plane, producing layered structures (Figure 2C). Because the interface regions observed in Form I contain the X/X’ and valine CH(CH_3_)_2_ groups, there is sufficient space to accommodate the imposed structural differences of the X/X’ groups. The ability of Form I to promote quasiracemate formation is apparent when considering that all targeted quasienantiomeric components achieve quasiracemate formation with **1**-CF_3_. These quasiracemates include the H/CF_3_ (%Δ*V* = 139%), F/CF_3_ (100), CH_3_/CF_3_ (59), Cl/CF_3_ (56), Br/CF_3_ (40), NO_2_/CF_3_ (27), I/CF_3_ (20) and *t*-Bu/CF_3_ (40) pairings with %Δ*V* ranging from 20 to 139.

**Figure 3:**
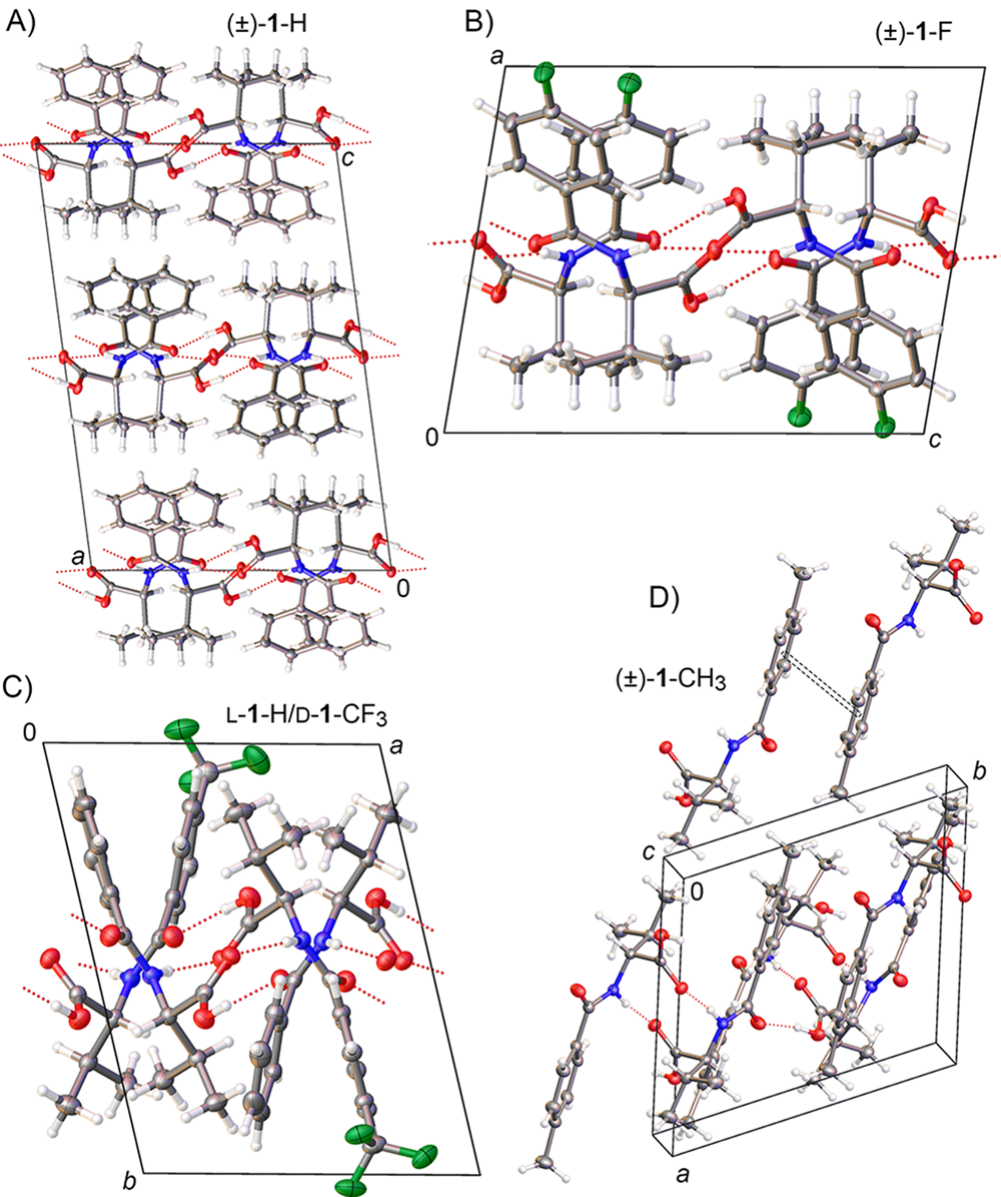
Crystal structure packing diagrams of (A) (±)-**1**-H, (B) (±)-**1**-F, (C) l-**1**-H/d-**1**-CF_3_, and (D) (±)-**1**-CH_3_ showing unit cell contents and the N–H···O_amide_*R*_2_^2^(10) and O–H···O_carboxyl_ C(7) contacts.

The crystal structures of the (±)-**1**-H, (±)-**1**-F, (±)-**1**-CH_3_, and l-**1**-H/d-**1**-CF_3_ systems show different molecular packing from those aligned using Form I. Each of these four structures consists of two symmetry-independent molecules (*Z*’ = 2) organized in space groups *P*1 (l-**1**-H/d-**1**-CF_3_), *P*1̅ ((±)-**1**-F, (±)-**1**-CH_3_), or *P*2_1_/*c* ((±)-**1**-H) (Figure 3). While the *R*_2_^2^(10) and C(7) hydrogen-bond patterns offer consistent features with Form I, the relative orientation of the second component in the asymmetric unit can be different. Within this set of structures, the (±)-**1**-H and (±)-**1**-F systems show many similar packing features despite different unit cell and space group settings. The observed difference is noticeable for l-**1**-H/d-**1**-CF_3_ and (±)-**1**-CH_3_, where (±)-**1**-CH_3_ provides the only structure exhibiting π-stacking with close aryl–aryl interplanar (3.447 Å), centroid-centroid (3.622 Å), and aryl–aryl shift (1.365 Å) distances.

The structural control promoted by Form I is evident when further considering the H, F, and CH_3_ components. The racemic structures produced from these H, F, and CH_3_ materials crystallize with molecular alignment different from Form I packing observed in the other racemates (i.e., Cl, Br, NO_2_, I, CF_3_, and *t*-Bu). Interestingly, cocrystallizing l-**1**-CF_3_ with the H, F, and CH_3_ quasienantiomers produced the F/CF_3_ and CH_3_/CF_3_ quasiracemates, each taking on Form I alignment. However, from pairing the H and CF_3_ quasienantiomers, the structural disparity of these components is sufficient to create a different crystal alignment than the other 16 racemates and quasiracemates.

**Figure 4:**
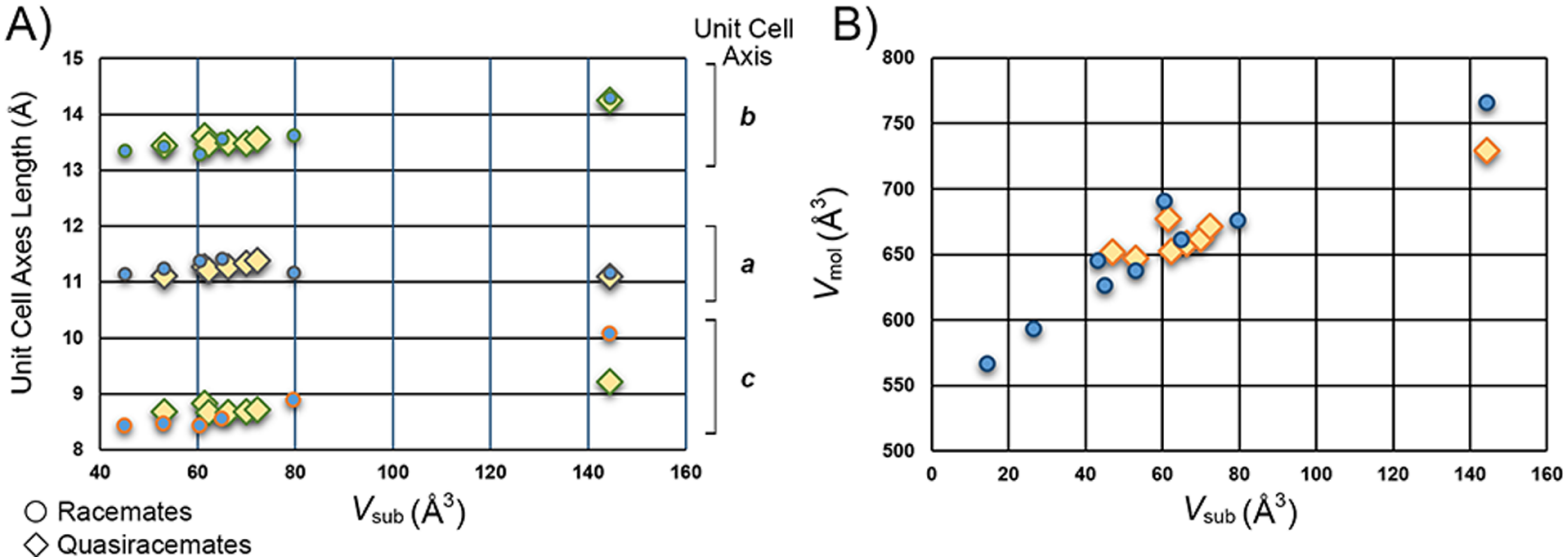
Plots of substituent volumes (*V*_sub_) and (A) crystallographic unit cell axes of Form I and (B) racemic or quasiracemic pair volumes (*V*_mol_) for all benzoyl valine **1** structures.

Given the hydrogen-bond and crystal packing similarities for the 17 racemates and quasiracemates, we wondered if the spatial features of X and X’ relate directly to the molecular volumes and unit cell parameters. Inspection of Figure 4A shows Form I structures and a close relationship between their substituent volumes (*V*_sub_ = *V*_X_ + *V*_X’_, where X and X’ represent racemic and quasiracemic functional group pairs) and the length of the unit cell axes. This data offer yet another indication of the isomorphic behavior of Form I. Figure 4B compares *V*_sub_ for all structures to the volume of the racemic or quasiracemic molecular pair (*V*_mol_ = unit cell volume/number of unit cell racemic or quasiracemic molecular pairs) and indicates a strong correlation between the *V*_sub_ and. *V*_mol_ data.

Further investigating the isomorphic nature of these crystal structures, we then turned our attention to the Crystal Structure Similarity facility found in the Materials section of CCDC Mercury. This structural tool provides a quantitative measure of the structural similarity for sets of crystal structures.^2,46^ The data provided in Table 1 was prepared by comparing clusters of 15 molecules with a 20% tolerance for distances and angles. This search permitted chemical differences, inverted structures when appropriate, and included hydrogen atoms in the calculations. Each data cell corresponds to a crystal structure pair and provides information about the degree of fit using a root-mean-square (RMS) fit approach and the number of common molecules within the specified search constraints. Data cells with low RMS values for clusters of more than ten molecules indicate highly correlated structures. Though visually inspecting sets of crystal packing patterns and unit cell parameters can provide a reasonable first approach to crystal structure assessment, this CCDC Mercury search tool offers a powerful method for generating large data sets for evaluation. As shown in Table 1, the structures corresponding to Form I correlate well. For example, (±)-**1**-CF_3_ produces crystal packing similar to the other structures assigned to Form I, as evidenced by RMS ranges of 0.18 to 0.74 with 9 to 15 molecules in the clusters.

**Table 1:**
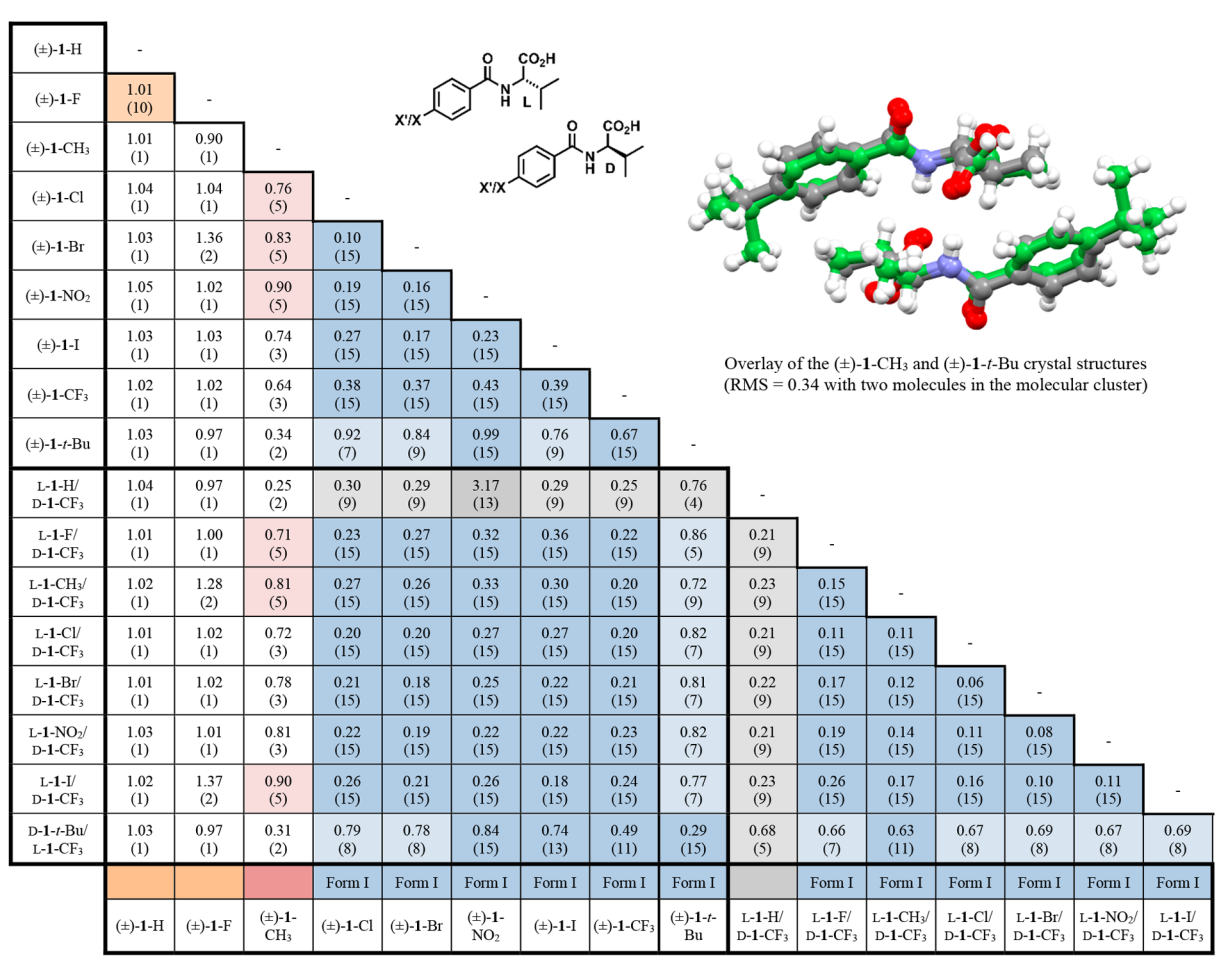
CCDC Mercury Crystal Packing Similarity Assessments of the 17 Benzoyl Valine **1** Racemic and Quasiracemic Structures^a^

a Cell values indicate root-mean-square agreement of the structural pair with the number of common molecules provided in parentheses (maximum of 15 molecules). The light- and dark-shaded cells indicate entries with 4–9 and 10–15 molecules in the cluster, respectively.

The structures with *t*-butyl groups (*i.e.*, (±)-**1**-CF_3_ and d-**1**-*t*-Bu/l-**1**-CF_3_) deviate somewhat from the other Form I systems, where 16 of the thirty-two entries indicate clusters of 5–9 molecules within the search limits. This diminished correlation is not due to orientation differences but to molecular spacing arising from the bulky *t*-butyl groups. Other significant findings include the structure similarity of the (±)-**1**-H and (±)-**1**-F entries despite their unit cell and space group differences (RMS = 1.01 with ten molecules included in the cluster). The packing patterns for these materials are distinct from the other 15 structures. Also, quasiracemate l-**1**-H/d-**1**-CF_3_ shows a notable correlation to other Form I entries (0.76(4 molecules) to 3.17(13 molecules)) and (±)-**1**-CH_3_ has only a modest level of common structural packing features with the other structures as determined by fewer than six molecules in the correlated molecular clusters.

### Conformational Analysis

**Figure 5:**
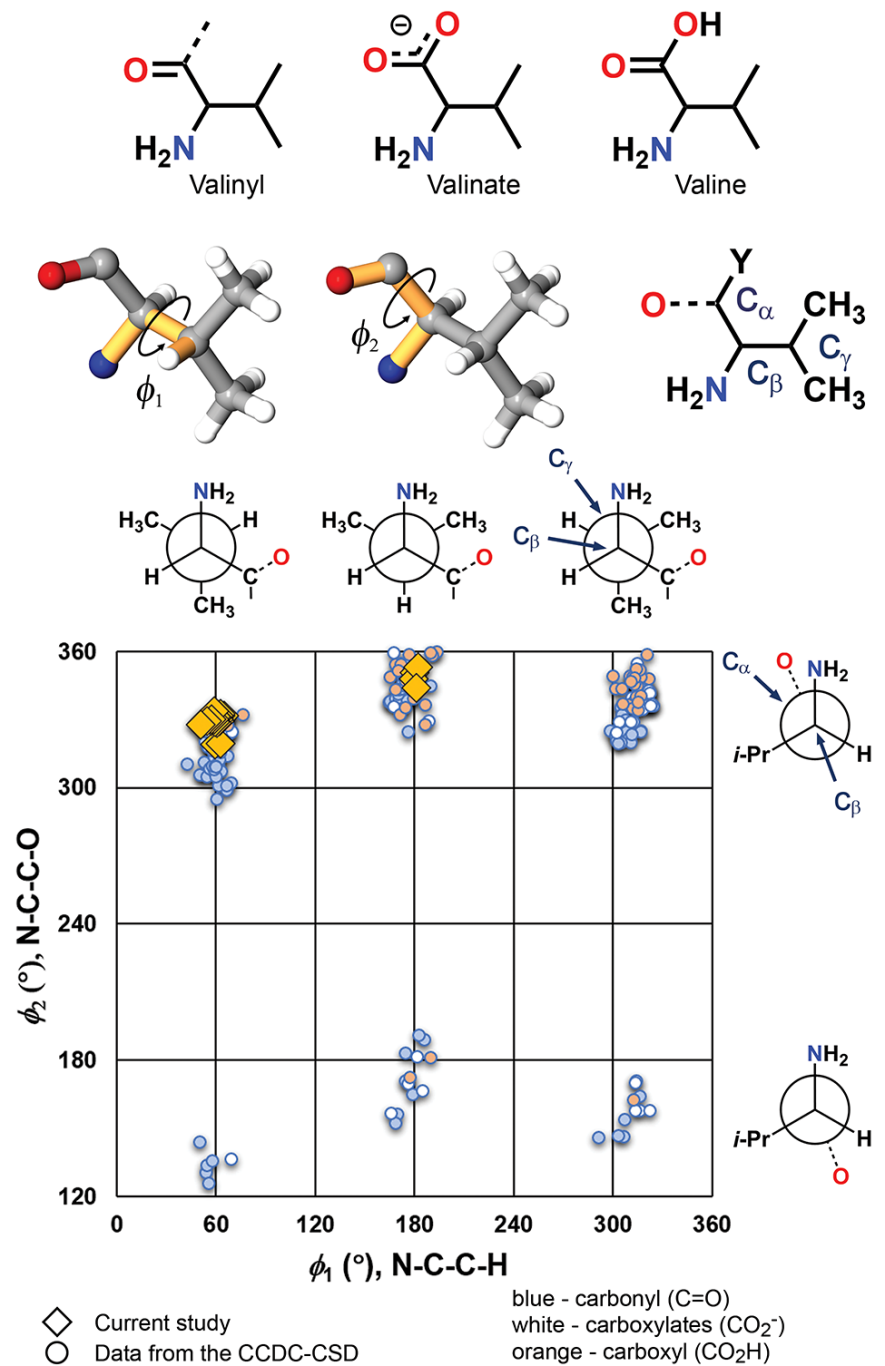
CSD search for valinyl, valinate, and valine fragments showing a 2D plot of the N–C–C–H (ϕ_1_) and N–C–C–O (ϕ_2_) dihedral angles.

The valine fragments found in this study and those from the extant crystallographic database were examined to identify the role that crystal packing plays in directing the component topological features of these systems. A CCDC Cambridge Structural Database^1^ (CSD, vs 5.43) search for valinyl, valinate, and valine fragments retrieved 99 (55 refcodes), 47 (39 refcodes), and 52 (32 refcodes) hits, respectively, and were compared to the benzoyl valine **1** structures (Figure 5). When necessary, the structures were inverted to the *S* isomer to ensure consistency of the stereochemical configurations. Combining this data with the current study (17 structures, 30 unique molecules) gave 228 distinct valine fragments. Dihedral angle (ϕ) distributions are presented in Figure 5 as 2D plots. A comparison of the N–C_β_-C_γ_-H (ϕ_1_) and N–C_β_-C_α_-O (ϕ_2_) dihedral angles shows data clusters with ϕ_1_ near 60, 180, and 310° and ϕ_2_ close to 160° and 320°. While the grouping of ϕ_1_ values likely arises from steric repulsions of the −CH(CH_3_)_2_ group, the structural preferences of the ϕ_2_ data are somewhat surprising given the potential structural diversity of the valine derivatives represented in this complete data set. Even so, this carboxyl and carboxylate ratcheting effect in valine and other amino acids was identified as early as the 1970s.^47,48^

The ϕ_1_ and ϕ_2_ values corresponding to the valinyl, valinate, and valine fragments show noticeably similar conformational landscapes (Figure 5). Each of these search queries lacks entries with N–C–C–O angles (ϕ_2_) < 100° and populate the six remaining bins (40° < ϕ_1_ < 90°, 160° < ϕ_1_ < 200°, 290° < ϕ_1_ < 320°, 120° < ϕ_2_ < 190°, 290° < ϕ_2_ < 360°). The 17 structures from this study (30 unique entries) adopt closely related conformations and populate two bins with ϕ_1_ < 190° and ϕ_2_ > 310°. This structural consistency follows the observed isomorphism, with the (±)-**1**-H and (±)-**1**-F systems deviating from this structural trend where ϕ_1_ ∼ 180° and ϕ_2_ ∼ 350°.

An early report describes the likelihood of valine displaying one of the three isopropyl rotamers as nearly equal.^47^ More recently, Lessasi et al. applied rotational spectroscopy to neutral valine and showed the relative stability of the conformers as ϕ_1_ (310°, 4.3 kJ/mol) > ϕ_1_ (180°, 2.3 kJ/mol) > ϕ_1_ (60°, 0.0 kJ/mol).^49^ Because the energetically favored rotamer is not present in the benzoyl valine (**1**) systems, this likely indicates that crystal packing must play a substantial role in the orientation of the isopropyl groups.

### Lattice Energies

A theoretical approach to evaluating crystal lattice energies (*E*_Latt_) was pursued to understand the role of the racemic and quasiracemic components in the formation of the observed crystalline phases. Given the isomorphic nature of the family of benzoyl valine **1** compounds, it is expected that similar trends should exist with the *E*_Latt_ values. These computational efforts utilize the program Crystal Explorer^28,50^ (CE, Gaussian16,^29^ B3LYP/6-31G(dp)), start with the crystal structure coordinates obtained from the racemic and quasiracemic materials and sum the electrostatic, polarization, dispersion, and repulsion contributions to *E*_Latt_. Recently, the CE-B3LYP method was applied to extensive sets of crystallographic data to establish the confidence level for lattice energy calculations.^51^ Outcomes from this study revealed calculated E_Latt_ estimates comparable to those of databases of benchmark experimental lattice energies.

The *E*_Latt_ values in Table 2 support many of the observed crystal structure trends for the benzoyl valine **1** family. For the racemates, the computationally derived *E*_Latt_ values steadily increase from X = H to *t*-Bu, with subtle variances likely due to molar mass, where this trend is most evident with the isomorphic structures ranging from (±)-Cl to (±)-*t*-Bu. For the quasiracemates, similar changes exist where an increase in the *E*_Latt_ value corresponds to the material composition. Another important consideration is that comparable *E*_Latt_ energies were determined for each quasiracemate, and the related racemate supported the idea that quasiracemates mimic the thermodynamically preferred centrosymmetric alignment observed in the racemates. The Cl/CF_3_ quasiracemic system illustrates this relationship where the crystal lattice energies of the quasiracemate (*E*_Latt(Cl/CF_3_)_ = −161.1 kJ) and racemates (*E*_Latt(±-Cl)_ = −159.3 and *E*_Latt(±−CF_3_)_ = −163.6) with differences in these energies Δ*E*_Latt_ = 1.8 and 2.5 2 kJ/mol. The crystal density and E_Latt_ data related to these entries are quite similar and consistent with structures that exhibit closely related crystal packing.

**Table 2:** Crystal Lattice Enthalpies Derived from Crystal Explorer (B3LYP/6-31G(d,p), Gaussian 16) and Crystal Densities for Benzoyl Valines (**1**)

	crystal lattice enthalpies, *E*_Latt_ (kJ/mol)	density (g/mL)
(±)-**1**-H	–146.5	1.297
(±)-**1**-F	–144.1	1.339
(±)-**1**-CH_3_	–163.2	1.211
(±)-**1**-Cl	–159.3	1.356
(±)-**1**-Br	–166.2	1.563
(±)-**1**-NO_2_	–172.9	1.388
(±)-**1**-I	–184.2	1.744
(±)-**1**-CF_3_	–163.6	1.421
(±)-**1**-*t*-Bu	–184.5	1.203
l-**1**-H/d-**1**-CF_3_	–155.7	1.300
l-**1**-F/d-**1**-CF_3_	–155.8	1.356
l-**1**-CH_3_/d-**1**-CF_3_	–159.3	1.286
l-**1**-Cl/d-**1**-CF_3_	–161.1	1.386
l-**1**-Br/d-**1**-CF_3_	–162.8	1.486
l-**1**-NO_2_/d-**1**-CF_3_	–167.9	1.393
l-**1**-I/d-**1**-CF_3_	–179.1	1.574
d-**1**-*t*-Bu/l-**1**-CF_3_	–175.2	1.290

This computational investigation also compared the benzoyl valines to diarylamide **2**, naphthylamide **3** and benzoyl leucine/phenyl **4** to understand the effect of the chemical framework on crystal stabilization. Our previous report showed that Δ*E*_Latt_ (**2**, **3**) is ∼20 kJ/mol in favor of naphthylamide **3** derivatives. Given the availability, the racemic X = H derivatives for the **1**, **3** and **4** systems provide a common substituent for assessing the relative crystal stabilities of these materials. The difference in CE lattice energy for the (±)-**1**-H and (±)-**3**-H (*E*_Latt_ = −92.2 kJ/mol), (±)-**4**-Leu-H (−161.5 kJ/mol) and (±)-**4**-Phe-H (−182.9 kJ/mol) systems is Δ*E*_Latt_ = −54.3, + 15.0 and +36.4 kJ/mol, respectively. These results show that crystal lattice stabilization favors **1** when compared to naphthylamide **3**; however, the leucine and phenylalanine **4** racemates, structures with the same hydrogen bond capacity as **1** but occupying a larger molecular space, create more thermodynamically preferred crystal structures. This combined data provides considerable support that larger molecular architectures capable of creating substantial nonbonded contact networks can promote greater structural diversity of quasienantiomeric components during molecular recognition events.

## Conclusions

The crystal structures for a family of benzoyl valine (**1**) analogs show a high success rate of quasiracemate formation due to the complementary topologies of the quasienantiomeric components. A more complete view of the molecular recognition landscape was achieved by combining a 1:1 ratio of **1**-CF_3_ with a diverse set of quasienantiomeric components, where X = H, F, CH_3_, Cl, Br, NO_2_, I, and *t*-butyl. Given the extent of their shape-space differences, many of these functional group pairs were previously considered unlikely. In light of the other known small-molecule quasiracemates, the results from this report are particularly striking, since all systems examined formed quasiracemates. Many of the crystal structures of these materials and their racemic counterparts display a high degree of isomorphism, with the components aligned into *R*_2_^2^(10) and C(7) hydrogen-bond motifs. CSD and crystal structure similarity searches, conformational assessments, and lattice energy determinations point to the benzoyl amino acid systems as exemplary molecular frameworks for examining the quasiracemate behavior. Such cocrystalline systems have the potential for various applications where molecular chirality and controlled motifs offer access to prescribed supramolecular asymmetry.

## Supporting Information Available

Additional experimental details including NMR data, crystallographic data and hydrogen-bond parameters (PDF)

## Abbreviations

CCDC: Cambridge Crystallographic, Data Centre
CE: Crystal Explorer
CSD: Cambridge Structural Database
