# Supplementary material for: Benzoyl Valine Quasiracemates: Pairing CF3 Quasienantiomers with H to t-Butyl
Source: Cryst Growth Des. 2024 Apr 15;24(9):3967–76. doi: 10.1021/acs.cgd.4c00307 (PMC11066836; doi:10.1021/acs.cgd.4c00307)
Supplement: Supplementary file 1 — cg4c00307_si_001.pdf [file cg4c00307_si_001.pdf]

Electronic Supporting Information for:

Benzoyl Valine Quasiracemates: Pairing CF_3 Quasienantiomers with H to *t*-Butyl

Ashah M. Gould, Danielle R. Schalk, Molly E. Fleagle and Kraig A. Wheeler*

Department of Chemistry, Whitworth University, 300 West Hawthorne Road, Spokane, Washington, 99251, USA. Email: kraigwheeler@whitworth.edu.

Table of Contents

SI 1. Spectroscopic Data

SI 2. Crystallographic Data

SI 1. Spectroscopic Data for Benzoyl Valine 1.

General Considerations

^{1}H NMR spectral data were recorded with a 400 MHz JEOL 400SS spectrometer using the Delta software (4.3.6.0). Spectral data are referenced using the solvent residual signal as an internal standard, and chemical shift values are expressed as δ values (ppm) and the value of coupling constants (*J*) in Hertz (Hz). The following abbreviations were used for signal multiplicities: s, singlet; d, doublet; dd, doublet of doublets; t, triplet; q, quartet; m, multiplet; and br, broad.

NMR Spectoscopic Data

N-benzoyl-L-valine, L-**1**-H

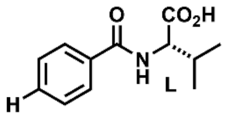

^{1}H-NMR ($CDCl_3$): δ 7.81 (d, *J* = 8.6 Hz, 2H, C_{Ar}-H); 7.55-7.42 (m, 3 H, C_{Ar}-H); 6.71 (d, *J* = 8.7 Hz, 1H, N-H); 4.80 (dd, *J* = 5.0 and 8.7 Hz, 1H, C_{sp3}-H); 2.41-2.31 (m, 3H, CH_3); 1.06 (d, *J* = 6.7 Hz, 3H, CH_3); 1.02 (d, *J* = 6.7 Hz, 3H, CH_3).

N-benzoyl-D-valine, D-**1**-H

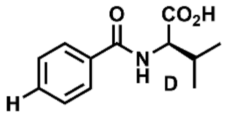

^{1}H-NMR ($CDCl_3$): δ 7.80 (d, *J* = 8.7 Hz, 2H, C_{Ar}-H); 7.54-7.42 (m, 3 H, C_{Ar}-H); 6.72 (d, *J* = 8.7 Hz, 1H, N-H); 4.80 (dd, *J* = 5.0 and 8.7 Hz, 1H, C_{sp3}-H); 2.41-2.31 (m, 3H, CH_3); 1.05 (d, *J* = 6.6 Hz, 3H, CH_3); 1.03 (d, *J* = 6.6 Hz, 3H, CH_3).

N-(*p*-fluorobenzoyl)-L-valine, L-**1**-F

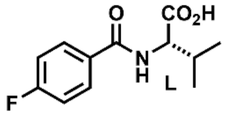

^{1}H-NMR ($CDCl_3$): δ 7.82 (d, *J* = 8.2 Hz, 2H, C_{Ar}-H); 7.12 (d, *J* = 8.2Hz, 1H, C_{Ar}-H); 6.62 (d, *J* = 8.7 Hz, 1H, N-H); 4.78 (dd, *J* = 5.3 and 8.7 Hz, 1H, C_{sp3}-H); 2.43-2.33 (s, 3H, CH_3); 1.05 (d, *J* = 6.7 Hz, 3H, CH_3); 1.03 (d, *J* = 6.7 Hz, 3H, CH_3).

N-(*p*-fluorobenzoyl)-D-valine, D-**1**-F

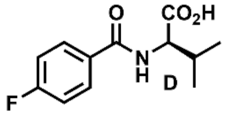

^{1}H-NMR ($CDCl_3$): δ 7.82 (d, *J* = 8.2 Hz, 2H, C_{Ar}-H); 7.12 (d, *J* = 8.2Hz, 1H, C_{Ar}-H); 6.62 (d, *J* = 8.7 Hz, 1H, N-H); 4.78 (dd, *J* = 5.3 and 8.7 Hz, 1H, C_{sp3}-H); 2.43-2.33 (s, 3H, CH_3); 1.05 (d, *J* = 6.7 Hz, 3H, CH_3); 1.03 (d, *J* = 6.7 Hz, 3H, CH_3).

N-(*p*-methylbenzoyl)-L-valine, L-**1**-CH_3

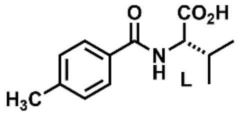

^{1}H-NMR ($CDCl_3$): δ 7.64 (d, *J* = 8.2 Hz, 2H, C_{Ar}-H); 7.16 (d, *J* = 8.2Hz, 1H, C_{Ar}-H); 6.80 (d, *J* = 8.0 Hz, 1H, N-H); 4.63 (dd, *J* = 5.4 and 8.0 Hz, 1H, C_{sp3}-H); 2.41-2.31 (m, 1H, C_{sp3}-H); 0.95 (d, *J* = 6.3 Hz, 3H, CH_3); 0.91 (d, *J* = 6.3 Hz, 3H, CH_3).

N-(*p*-methylbenzoyl)-D-valine, D-**1**-CH_3

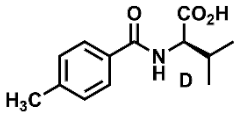

^{1}H-NMR ($CDCl_3$): δ 7.65 (d, *J* = 8.2 Hz, 2H, C_{Ar}-H); 7.14 (d, *J* = 8.2Hz, 1H, C_{Ar}-H); 6.81 (d, *J* = 7.9 Hz, 1H, N-H); 4.66 (dd, *J* = 5.3 and 7.9 Hz, 1H, C_{sp3}-H); 2.41-2.31 (m, 1H, C_{sp3}-H); 0.93 (d, *J* = 6.2 Hz, 3H, CH_3); 0.91 (d, *J* = 6.2 Hz, 3H, CH_3).

N-(*p*-chlorobenzoyl)-L-valine, L-**1**-Cl

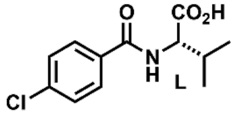

^{1}H-NMR ($CDCl_3$): δ 7.71 (d, *J* = 8.2 Hz, 2H, C_{Ar}-H); 7.39 (d, *J* = 8.2 Hz, 1H, C_{Ar}-H); 6.69 (d, *J* = 8.3 Hz, 1H, N-H); 4.74 (dd, *J* = 5.0 and 8.3 Hz, 1H, C_{sp3}-H); 2.41-2.31 (m, 1H, C_{sp3}-H); 1.02 (d, *J* = 6.8 Hz, 3H, CH_3); 1.00 (d, *J* = 6.8 Hz, 3H, CH_3).

N-(*p*-chlorobenzoyl)-D-valine, D-**1**-Cl

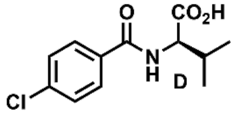

^{1}H-NMR ($CDCl_3$): δ 7.68 (d, *J* = 8.2 Hz, 2H, C_{Ar}-H); 7.34 (d, *J* = 8.2 Hz, 1H, C_{Ar}-H); 6.93 (d, *J* = 8.7 Hz, 1H, N-H); 4.68 (dd, *J* = 5.1 and 8.7 Hz, 1H, C_{sp3}-H); 2.41-2.31 (m, 1H, C_{sp3}-H); 1.00 (d, *J* = 6.7 Hz, 3H, CH_3); 0.98 (d, *J* = 6.7 Hz, 3H, CH_3).

N-(*p*-bromobenzoyl)-L-valine, L-**1**-Br

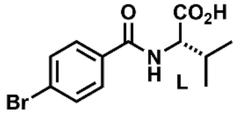

^{1}H-NMR ($CDCl_3$): δ 7.67 (d, *J* = 8.1 Hz, 2H, C_{Ar}-H); 7.59 (d, *J* = 8.1 Hz, 1H, C_{Ar}-H); 6.67 (d, *J* = 8.7 Hz, 1H, N-H); 4.79 (dd, *J* = 4.8 and 8.7 Hz, 1H, C_{sp3}-H); 2.42-2.32 (m, 1H, C_{sp3}-H); 1.05 (d, *J* = 6.8 Hz, 3H, CH_3); 1.01 (d, *J* = 6.8 Hz, 3H, CH_3).

N-(*p*-bromobenzoyl)-D-leucine, D-**1**-Br

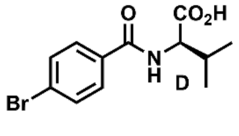

^{1}H-NMR ($CDCl_3$): δ 7.66 (d, *J* = 8.2 Hz, 2H, C_{Ar}-H); 7.59 (d, *J* = 8.2 Hz, 1H, C_{Ar}-H); 6.67 (d, *J* = 8.5 Hz, 1H, N-H); 4.78 (dd, *J* = 4.9 and 8.5 Hz, 1H, C_{sp3}-H); 2.42-2.32 (m, 1H, C_{sp3}-H); 1.03 (d, *J* = 6.8 Hz, 3H, CH_3); 1.02 (d, *J* = 6.8 Hz, 3H, CH_3).

N-(*p*-nitrobenzoyl)-L-valine, L-**1**-NO_2

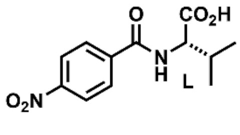

^{1}H-NMR ($CDCl_3$): δ 8.32 (d, *J* = 8.3 Hz, 2H, C_{Ar}-H); 7.98 (d, *J* = 8.3 Hz, 1H, C_{Ar}-H); 6.74 (d, *J* = 8.5 Hz, 1H, N-H); 4.83 (dd, *J* = 4.5 and 8.5 Hz, 1H, C_{sp3}-H); 2.45-2.35 (m, 1H, C_{sp3}-H); 1.08 (d, *J* = 6.5 Hz, 3H, CH_3); 1.04 (d, *J* = 6.5 Hz, 3H, CH_3).

N-(*p*-nitrobenzoyl)-D-phenylalanine, D-**1**-NO_2

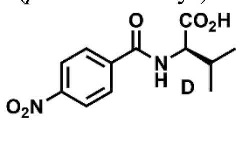

^{1}H-NMR ($CDCl_3$): δ 8.31 (d, *J* = 8.2 Hz, 2H, C_{Ar}-H); 7.96 (d, *J* = 8.2 Hz, 1H, C_{Ar}-H); 6.77 (d, *J* = 8.4 Hz, 1H, N-H); 4.83 (dd, *J* = 4.6 and 8.4 Hz, 1H, C_{sp3}-H); 2.45-2.35 (m, 1H, C_{sp3}-H); 1.05 (d, *J* = 6.5 Hz, 3H, CH_3); 1.02 (d, *J* = 6.5 Hz, 3H, CH_3).

N-(*p*-iodobenzoyl)-L-valine, L-**1**-I

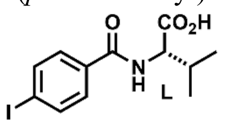

^{1}H-NMR ($CDCl_3$): δ 7.81 (d, *J* = 8.2 Hz, 2H, C_{Ar}-H); 7.53 (d, *J* = 8.2 Hz, 1H, C_{Ar}-H); 6.62 (d, *J* = 8.7 Hz, 1H, N-H); 4.79 (dd, *J* = 5.0 and 8.7 Hz, 1H, C_{sp3}-H); 2.41-2.31 (m, 1H, C_{sp3}-H); 1.05 (d, *J* = 6.7 Hz, 3H, CH_3); 1.01 (d, *J* = 6.6 Hz, 3H, CH_3).

N-(*p*-iodobenzoyl)-D-valine, D-**1**-I

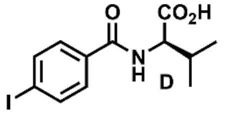

^{1}H-NMR ($CDCl_3$): δ 7.79 (d, *J* = 8.1 Hz, 2H, C_{Ar}-H); 7.51 (d, *J* = 8.1 Hz, 1H, C_{Ar}-H); 6.65 (d, *J* = 8.7 Hz, 1H, N-H); 4.74 (dd, *J* = 5.1 and 8.7 Hz, 1H, C_{sp3}-H); 2.41-2.31 (m, 1H, C_{sp3}-H); 1.04 (d, *J* = 6.7 Hz, 3H, CH_3); 1.02 (d, *J* = 6.7 Hz, 3H, CH_3).

N-(*p*-trifluoromethylbenzoyl)-L-valine, L-**1**-CF_3

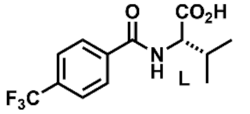

^{1}H NMR ($CDCl_3$): δ 7.97 (d, *J* = 7.8 Hz, 2H, C_{Ar}-H); 7.69 (d, *J* = 7.8Hz, 1H, C_{Ar}-H); 7.29 (d, *J* = 8.7 Hz, 1H, N-H); 4.66 (dd, *J* = 5.1 and 8.2 Hz, 1H, C_{sp3}-H); 2.35-2.25 (m, 1H, C_{sp3}-1H); 1.03 (d, *J* = 6.4 Hz, 3H, CH_3); 1.01 (d, *J* = 6.4 Hz, 3H, CH_3)

N-(*p*-trifluoromethylbenzoyl)-D-valine, D-**1**-CF_3

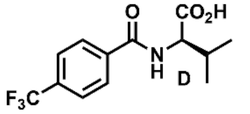

^{1}H-NMR ($CDCl_3$): δ 7.82 (d, *J* = 7.8 Hz, 2H, C_{Ar}-H); 7.60 (d, *J* = 7.8 Hz, 1H, C_{Ar}-H); 6.88 (d, *J* = 8.2 Hz, 1H, N-H); 4.64 (dd, *J* = 5.5 and 8.2 Hz, 1H, C_{sp3}-H); 2.35-2.25 (m, 1H, C_{sp3}-H) 0.99 (d, *J* = 6.3 Hz, 3H, CH_3); 0.97 (d, *J* = 6.3 Hz, 3H, CH_3).

N-(*p*-*t*-butylbenzoyl)-L-valine, L-**2**-*t*-Bu

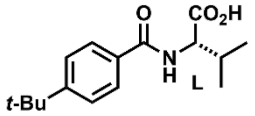

^{1}H-NMR ($CDCl_3$): δ 7.76 (d, *J* = 8.2 Hz, 2H, C_{Ar}-H); 7.49 (d, *J* = 8.2 Hz, 1H, C_{Ar}-H); 6.62 (d, *J* = 8.1 Hz, 1H, N-H); 4.79 (dd, *J* = 5.2 and 8.1 Hz, 1H, C_{sp3}-H); 2.41-2.31 (m, 1H, C_{sp3}-H); 1.34 (s, 9H, CH_3); 1.06 (d, *J* = 6.3 Hz, 3H, CH_3); 1.03 (d, *J* = 6.3 Hz, 3H, CH_3).

N-(*p*- *t*-butylbenzoyl)-D-valine, D-**2**-*t*-Bu

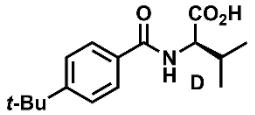

^{1}H-NMR ($CDCl_3$): δ 7.78 (d, *J* = 8.2 Hz, 2H, C_{Ar}-H); 7.50 (d, *J* = 8.2 Hz, 1H, C_{Ar}-H); 6.62 (d, *J* = 8.2 Hz, 1H, N-H); 4.76 (dd, *J* = 5.2 and 8.2 Hz, 1H, C_{sp3}-H); 2.41-2.31 (m, 1H, C_{sp3}-H); 1.35 (s, 9H, CH_3); 1.06 (d, *J* = 6.4 Hz, 3H, CH_3); 1.03 (d, *J* = 6.4 Hz, 3H, CH_3).

SI 2. Crystallographic Data

Table S1. Crystallographic Data for Racemic and Quasiracemic Benzoyl Valine **1**.

	(±)-**1**-H	(±)-**1**-F	(±)-**1**-CH_3	(±)-**1**-Cl
Crystal data				
CCDC deposit no.	2334389	2334386	2334380	2334379
Empirical formula	$C_{12}H_{15}NO_3$	$C_{12}H_{14}FNO_3$	$C_{13}H_{17}NO_3$	$C_{12}H_{14Cl}NO_3$
Crystal System, space group	Monoclinic *P*2$_1$/*c* (no. 14)	Monoclinic *P*-1 (no. 2)	Monoclinic *P*-1 (no. 2)	Monoclinic *P*2$_1$/*c* (no. 14)
M_r	221.25	239.24	235.27	255.69
a, Å	12.4353(9)	9.5893(4)	10.2096(6)	11.1482(3)
b, Å	10.3068(7)	10.3590(4)	10.5015(6)	8.4268(2)
c, Å	17.8334(13)	12.2544(5)	13.7292(8)	13.3480(4)
α, deg	90	88.293(2)	90.500(2)	90
β, deg	97.490(3)	80.487(2)	109.789(2)	92.799(1)
γ, deg	90	81.229(2)	109.790(13)	90
V, (Å^3)	2266.2(3)	1186.51(8)	1290.55(12)	1252.46(6)
Z, *Z'*	8, 2	4, 2	4, 2	4, 1
D_{calc} (g cm^{-3})	1.297	1.339	1.211	1.356
Radiation	Cu	Cu	Cu	Cu
μ (mm^{-1}), Cu $K\alpha$	0.767	0.900	0.702	2.687
F_{000}	944	504	504	536
temp (K)	100(2)	100(2)	100(2)	100(2)
Crystal form, color	prism, colorless	plate, colorless	plate, colorless	plate, colorless
Crystal size, mm	0.30x0.23x0.11	0.40x0.30x0.06	0.17x0.111x0.03	0.19x0.07x0.02
Data collection				
Diffractometer	Bruker Venture D8 CCD	Bruker Venture D8 CCD	Bruker Venture D8 CCD	Bruker Venture D8 CCD
T_{min}/ T_{max}	0.753/0.686	0.754/0.676	0.753/0.640	0.753/0.641
No. of refls. (meas., uniq., and obs.)	37502/4151/3983	20571/4653/4335	33896/2907/2782	9417/2287/2006
R_{int}	0.0414	0.0252	0.0354	0.0337
θ_{max} (°)	68.28	67.679	68.323	68.281
Refinement				
R/R^2_ω (obs data)	0.0455/0.1144	0.0319/0.0841	0.0364/0.0956	0.0308/0.0758
R/R^2_ω (all data)	0.0459/0.1150	0.0342/0.0864	0.0420/0.1002	0.0370/0.0808
S	1.188	1.047	1.068	1.056
No. of refls.	4151	4653	2907	2287
No. of parameters	305	323	325	208
$\Delta\rho_{max/min}$ (e·Å^{-3})	0.406/-0.246	0.330/-0.189	0.218/-0.255	0.256/-0.203
flack	-	-	-	-

Table S1. Crystallographic Data for Racemic and Quasiracemic Benzoyl Valine **1**. (cont.)

	(±)-**1**-Br	(±)-**1**-NO_2	(±)-**1**-I	(±)-**2**-CF_3
Crystal data				
CCDC deposit no.	2334382	21kaw31	2334381	2334382
Empirical formula	$C_{12}H_{14}BrNO_3$	$C_{12}H_{14}N_2O_5$	$C_{12}H_{14}INO_3$	$C_{13}H_{14}F_3NO_3$
Crystal System, space group	Monoclinic $P2_1/c$ (no. 14)	Monoclinic $P2_1/c$ (no. 14)	Monoclinic $P2_1/n$ (no. 14)	Monoclinic $P2_1/c$ (no. 14)
M_r	300.15	266.25	347.14	289.20
a, Å	11.2445(9)	11.3824(3)	11.4220(19)	11.1730(3)
b, Å	8.4623(7)	8.4332(2)	8.5546(14)	8.8850(2)
c, Å	13.4251(11)	13.2950(3)	13.567(2)	13.6240(4)
α, deg	90	90	90	90
β, deg	93.460(3)	93.297(1)	94.171(6)	91.578(2)
γ, deg	90	90	90	90
V, (Å^3)	1275.13(18)	1381.12(5)	1322.1(4)	1351.97(6)
Z, Z'	4, 1	4, 1	4, 1	4, 1
D_{calc} (g cm^{-3})	1.563	1.388	1.744	1.421
Radiation	Cu	Cu	Mo	Cu
μ (mm^{-1}), Cu $K\alpha$	4.383	0.927	0.712	1.111
F_{000}	608	560	680	600
temp (K)	100(2)	100(2)	100(2)	100(2)
Crystal form, color	needle, colorless	plate, colorless	plate, colorless	plate, colorless
Crystal size, mm	0.22x0.13x0.03	0.42x0.18x0.012	0.38x0.11x0.07	0.32x0.18x0.05
Data collection				
Diffractometer	Bruker Venture D8 CCD	Bruker Venture D8 CCD	Bruker Venture D8 CCD	Bruker Venture D8 CCD
T_{min}/ T_{max}	0.754/0.573	0.754/0.618	0.745/0.624	0.753/0.652
No. of refls. (meas., uniq., and obs.)	18799/2520/2478	28521/2515/2183	20356/2429/2246	22602/2473/2256
R_{int}	0.0298	0.0469	0.0328	0.0357
θ_{max} (°)	72.36	72.028	25.388	68.246
Refinement				
R/R^2_ω (obs data)	0.0206/0.0519	0.0355/0.0897	0.0151/0.0359	0.0298/0.0748
R/R^2_ω (all data)	0.0210/0.0521	0.0420/0.0949	0.0176/0.0369	0.0333/0.07776
S	1.064	1.031	1.033	1.042
No. of refls.	2520	2515	2429	2473
No. of parameters	162	180	162	210
$\Delta\rho_{max/min}$ (e·Å^{-3})	0.339/-0.435	0.206/-0.238	0.319/-0.322	0.199/-0.244
flack	-	-	-	-

Table S1. Crystallographic Data for Racemic and Quasiracemic Benzoyl Valine **1**. (cont.)

	(±)-**1**-*t*-But	L-**1**-H/D-**1**-CF$_3$	L-**1**-F/D-**1**-CF$_3$	L-**1**-CH$_3$/D-**1**-CF$_3$
Crystal data				
CCDC deposit no.	2334387	2334392	2334378	2334390
Empirical formula	$C_{16}H_{23}NO_3$	$C_{25}H_{29}F_3N_2O_6$	$C_{25}H_{28}F_4N_2O_6$	$C_{26}H_{31}F_3N_2O_6$
Crystal System, space group	Monoclinic $P2_1/c$ (no. 14)	Monoclinic $P1$ (no. 1)	Monoclinic $P2_1$ (no. 4)	Monoclinic $P2_1/c$ (no. 14)
M_r	277.35	510.50	528.49	534.53
a, Å	11.1730(4)	8.7357(5)	11.1081(10)	11.2682(10)
b, Å	9.2868(3)	12.9973(7)	8.6743(8)	8.8278(8)
c, Å	14.7663(5)	13.3903(7)	13.4315(9)	13.6192(16)
α, deg	90	61.905(2)	90	90
β, deg	91.520(1)	89.016(2)	90.967(3)	91.178(5)
γ, deg	90	77.677(2)	90	90
V, (Å^3)	1531.63(9)	1304.16(13)	1294.01(19)	1354.5(2)
Z, Z'	4, 1	4, 2	2, 1	2, 1
D_{calc} (g cm^{-3})	1.203	1.300	1.356	1.286
Radiation	Cu	Cu	Mo	Cu
μ (mm^{-1}), Cu $K\alpha$	0.663	0.909	0.115	0.889
F_{000}	600	536	552	552
temp (K)	100(2)	100(2)	100(2)	100(2)
Crystal form, color	plate, colorless	plate, colorless	plate, colorless	needle, colorless
Crystal size, mm	0.28x0.16x0.05	0.36x0.11x0.08	0.29x0.29x0.14	0.43x0.25x0.09
Data collection				
Diffractometer	Bruker Venture D8 CCD	Bruker Venture D8 CCD	Bruker Venture D8 CCD	Bruker Venture D8 CCD
T_{min}/ T_{max}	0.754/0.665	0.754/0.662	0.745/0.570	0.754/0.667
No. of refls. (meas., uniq., and obs.)	21144/3011/2776	61513/9930/9690	21511/2844/2471	35516/5313/5281
R_{int}	0.0357	0.0393	0.0454	0.0251
θ_{max} (°)	72.07	72.336	26.442	73.391
Refinement				
R/R^2_{ω} (obs data)	0.0344/0.0862	0.0306/0.0846	0.0480/0.1244	0.0267/0.0734
R/R^2_{ω} (all data)	0.0371/0.0882	0.0313/0.0852	0.0575/0.1300	0.0268/0.0735
S	1.066	1.043	1.092	1.034
No. of refls.	3011	9930	2844	5313
No. of parameters	192	681	350	351
$\Delta\rho_{max/min}$ (e·Å^{-3})	0.253/-0.193	0.229/-0.145	0.414/-0.338	0.244/-0.189
flack	-	0.00(3)	-0.1(12)	0.13(2)

Table S1. Crystallographic Data for Racemic and Quasiracemic Benzoyl Valine **1**. (cont.)

	L-**1**-Cl/D-**1**-CF_3	L-**1**-Br/D-**1**-CF_3	L-**1**-NO_2/D-**1**-CF_3	L-**1**-I/D-**1**-CF_3
Crystal data				
CCDC deposit no.	2334384	2334391	2334385	2334393
Empirical formula	$C_{25}H_{28}ClF_3N_2O_6$	$C_{25}H_{28}BrF_3N_2O6$	$C_{25}H_{28}F_3N_3O8$	$C_{25}H_{28}F_3IN_2O_6$
Crystal System, space group	Monoclinic *P*2_1 (no. 4)	Monoclinic *P*2_1 (no. 4)	Monoclinic *P*2_1 (no. 4)	Monoclinic *P*2_1 (no. 4)
M_r	544.94	589.40	555.50	636.39
a, Å	11.2031(6)	11.2730(4)	11.3348(6)	11.3810(5)
b, Å	8.6621(4)	8.6643(4)	8.6762(5)	8.7152(4)
c, Å	13.4590(8)	13.4961(5)	13.4729(7)	13.5541(7)
α, deg	90	90	90	90
β, deg	91.930(2)	92.307(2)	92.067(3)	92.791(2)
γ, deg	90	90	90	90
V, (Å^3)	1305.35(12)	1317.13(9)	1324.10(12)	1342.81(11)
Z, *Z'*	2, 1	2, 1	2, 1	2, 1
D_{calc} (g cm^{-3})	1.386	1.486	1.393	1.574
Radiation	Mo	Cu	Cu	Cu
μ (mm^{-1}), Cu $K\alpha$	0.210	2.692	1.014	9.924
F_{000}	568	604	580	640
temp (K)	100(2)	100(2)	100(2)	100(2)
Crystal form, color	plate, colorless	needle, colorless	plate, colorless	needle, colorless
Crystal size, mm	0.25x0.20x0.08	0.37 x 0.11 x 06	0.16x0.10x0.04	0.39x0.04x0.03
Data collection				
Diffractometer	Bruker Venture D8 CCD	Bruker Venture D8 CCD	Bruker Venture D8 CCD	Bruker Venture D8 CCD
T_{min}/ T_{max}	0.745/0.641	0.754/0.579	0.754,0.591	0.753,0.463
No. of refls. (meas., uniq., and obs.)	19561/5306/4393	28206/5023/4781	28489/5128/3985	22571/4650/4449
R_{int}	0.0556	0.0442	0.0917	0.0474
θ_{max} (°)	26.409	72.138	72.321	68.514
Refinement				
R/R^2_ω (obs data)	0.0395/0.0838	0.0274/0.0665	0.0478/0.1276	0.0356/0.1114
R/R^2_ω (all data)	0.0562/0.0924	0.0299/0.0679	0.0647/0.1381	0.0385/0.1156
S	1.031	1.071	1.080	0.986
No. of refls.	5306	5023	5128	4650
No. of parameters	350	350	370	350
$\Delta\rho_{max/min}$ (e·Å^{-3})	0.238/-0.278	0.325/-0.525	0.258/-0.238	0.687/-0.572
flack	0.00(4)	0.013(6)	-0.25(15)	0.206(5)

Table S1. Crystallographic Data for Racemic and Quasiracemic Benzoyl Valine **1**. (cont.)

	D-**1**-*t*-But/L-**1**-CF_3
Crystal data	
CCDC deposit no.	2334394
Empirical formula	$C_{30}H_{33}ClN_2O_6$
Crystal System, space group	Monoclinic $P2_1$ (no. 4)
M_r	566.60
a, Å	11.1017(4)
b, Å	9.2197(4)
c, Å	14.2470(5)
α, deg	90
β, deg	90.466(2)
γ, deg	90
V, (Å^3)	1458.19(10)
Z, Z'	2, 1
D_{calc} (g cm^{-3})	1.290
μ (mm^{-1}), Cu $K\alpha$	0.863
F_{000}	600
temp (K)	100(2)
Crystal form, color	needle, colorless
Crystal size, mm	0.25x0.16x0.06
Data collection	
Diffractometer	Bruker Venture D8 CCD
T_{min}/ T_{max}	0.753,0.666
No. of refls. (meas., uniq., and obs.)	34770/5320/5213
R_{int}	0.0297
θ_{max} (°)	68.346
Refinement	
R/R^2_ω (obs data)	0.0331/0.0888
R/R^2_ω (all data)	0.0338/0.0895
S	1.040
No. of refls.	5320
No. of parameters	390
$\Delta\rho_{max/min}$ (e·Å^{-3})	0.384/-0.205
Flack	0.10(3)

Table S2. Hydrogen Bond Parameters for the Benzoyl Valine **1** Racemates and Quasiracemates.

Compound	Hydrogen Bond Type	D–H···A	D–H (Å)	H···A (Å)	D···A (Å)	D–H···A (°)	Symmetry operator
(±)-**1**-H		O2A–H2A···O1B	0.92(3)	1.70(3)	2.6201(18)	177(2)	-1+*x*, *y*, *z*
		N1A–H1A···O3A	0.87(2)	2.19(2)	3.026(2)	161(2)	-*x*, 0.5+*y*, 1.5-*z*
		O2B–H2B···O1A	0.93(2)	1.67(3)	2.5948(18)	173(2)	*x*, *y*, *z*
		N1B–H1B···O3B	0.86(2)	2.24(2)	3.065(2)	163(2)	1-*x*, 1.5-*y*, 1.5-*z*
(±)-**1**-F		O2A–H2A···O1B	0.870(17)	1.740(17)	2.6063(10)	173.1(15)	*x*, *y*, *z*
		N1A–H1A···O3A	0.861(15)	2.295(15)	3.1275(11)	162.5(12)	*1*-*x*, 2-*y*, 1-*z*
		O2B–H2B···O1A	0.903(16)	1.711(17)	2.6139(10)	178.3(15)	*x*, *y*, 1+*z*
		N1B–H1B···O3B	0.853(15)	2.242(15)	3.0729(11)	164.7(13)	1-*x*, 1-*y*, 2-*z*
(±)-**1**-CH_3		O2A–H2A···O1B	0.921(19)	1.677(15)	2.5875(13)	169.2(17)	*x*, *y*, *z*
		N1A–H1A···O3A	0.864(17)	2.040(14)	2.8973(14)	171.5(14)	1-*x*, 1-*y*, 2-*z*
		N1B–H1B···O3B	0.903(19)	2.012(14)	2.9064(14)	170.6(16)	1-*x*, 2-*y*, 1-*z*
		N1B–H1B···O3A	0.889(17)	1.727(14)	2.6039(12)	168.2(15)	*x*, 1+*y*, *z*
(±)-**1**-Cl	I	O2–H2···O1	0.83(29)	1.78(2)	2.6013(15)	173(2)	2-*x*, y+0.5, -0.5-*z*
		N1–H1···O3	0.816(19)	2.07(2)	2.8650(17)	166.2(17)	2-*x*, 1-*y*, -*z*
(±)-**1**-Br	I	O2–H2···O1	0.812(15)	1.791(16)	2.6014(14)	176(2)	1-*x*, *y*-0.5, 1.5-*z*
		N1–H1···O3	0.827(14)	2.055(14)	2.8662(15)	167.1(17)	1-*x*, 1-*y*, 1-*z*
(±)-**1**-NO_2	I	O2–H2···O1	0.85(2)	1.78(2)	2.6260(13)	175.7(18)	1-*x*, *y*-0.5, 1.5-*z*
		N1–H1···O3	0.868(16)	2.015(17)	2.8682(14)	167.2(14)	1-*x*, 1-*y*, 1-*z*
(±)-**1**-I	I	O2–H2···O1	0.815(15)	1.786(16)	2.5991(17)	176(2)	1-*x*, *y*+0.5, 1.5-*z*
		N1–H1···O3	0.822(14)	2.053(15)	2.8694(19)	172.3(19)	1-*x*, 1-*y*, 1-*z*
(±)-**1**-CF_3	I	O2–H2···O1	0.897(18)	1.698(18)	2.5951(12)	178.3(16)	1-*x*, *y*+0.5, 1.5-*z*
		N1–H1···O3	2.037(16)	2.037(16)	2.8674(13)	167.0(14)	1-*x*, 2-*y*, 1-*z*
(±)-**1**-*t*-Bu	I	O2–H2···O1	0.874(17)	1.744(17)	2.6152(11)	174.3(18)	1-*x*, *y*+0.5, 0.5-*z*
		N1–H1···O3	0.855(14)	2.108(15)	2.9582(12)	173.2(13)	1-*x*, 2-*y*, 1-*z*
L-**1**-H/D-**1**-CF_3		O2A–H2A···O1C	0.85(4)	1.78(4)	2.615(2)	168(3)	1-*x*, -*y*, -*z*
		N1A–H1A···O3B	0.85(3)	2.04(3)	2.869(2)	166(3)	*x*, *y*, *z*
		O2B–H2B···O1D	0.85(4)	1.78(4)	2.612(2)	173(3)	-1-*x*, -*y*, -*z*
		N1B–H1B···O3A	0.80(3)	2.11(3)	2.881(3)	164(3)	*x*, *y*, *z*
		O2C–H1C···O3A	0.87(3)	1.73(3)	2.604(2)	179(3)	*x*, *y*, *z*
		N1C–H1C···O3D	0.86(3)	2.04(3)	2.868(2)	162(3)	-1-*x*, *y*, -1-*z*
		O2D–H2D···O1B	0.87(4)	1.73(4)	2.603(2)	177(3)	*x*, *y*, *z*
		N1D–H1D···O3C	0.82(3)	2.07(3)	2.888(2)	172(3)	1-*x*, -*y*, 1-*z*
L-**1**-F/D-**1**-CF_3	I	O2A–H2A···O1A	0.85(2)	1.77(2)	2.618(4)	177(5)	1-*x*, -0.5+*y*, 1-*z*
		N1A–H1A···O3B	0.831(19)	2.04(2)	2.856(4)	166(4)	*x*, *y*, *z*
		O2B–H2B···O1B	0.837(19)	1.76(2)	2.578(4)	164(4)	1-*x*, 0.5+*y*, -*z*
		N1B–H1B···O3A	0.846(19)	2.03(2)	2.875(4)	173(4)	*x*, *y*, *z*
L-**1**-CH_3/D-**1**-CF_3	I	O2A–H2A···O1A	0.83(2)	1.81(2)	2.6327(19)	175(3)	1-*x*, 0.5+*y*, -*z*
		N1A–H1A···O3B	0.852(18)	2.054(19)	2.8896(19)	167(2)	*x*, *y*, *z*
		O2B–H2B···O1B	0.87(2)	1.77(2)	2.6417(19)	174(3)	1-*x*, -0.5+*y*, 1-*z*
		N1B–H1B···O3A	0.841(18)	2.072(19)	2.9058(19)	171(2)	*x*, *y*, *z*
L-**1**-Cl/D-**1**-CF_3	I	O2A–H2A···O1A	0.82(4)	1.78(4)	2.590(4)	169(4)	1-*x*, y+0.5, 2-*z*
		N1A–H1A···O3B	0.87(4)	2.00(3)	2.861(4)	170(4)	*x*, *y*, *z*
		O2B–H2B···O1B	0.90(4)	1.72(4)	2.615(4)	174(4)	1-*x*, -0.5+*y*, 1-*z*
		N1B–H1B···O3A	0.78(4)	2.10(3)	2.871(4)	168(4)	*x*, *y*, *z*
L-**1**-Br/D-**1**-CF_3	I	O2A–H2A···O1A	0.91(5)	1.68(5)	2.582(3)	169(4)	1-*x*, y+0.5, 2-*z*
		N1A–H1A···O3B	0.81(4)	2.06(4)	2.858(3)	168(4)	*x*, *y*, *z*
		O2B–H2B···O1B	0.86(4)	1.75(5)	2.607(3)	175(4)	1-*x*, 0.5+*y*, 1-*z*
		N1B–H1B···O3A	0.84(4)	2.05(4)	2.875(3)	167(4)	*x*, *y*, *z*
L-**1**-NO_2/D-**1**-CF_3	I	O2A–H2A···O1A	0.85(3)	1.77(3)	2.596(5)	164(5)	1-*x*, 0.5+*y*, -*z*
		N1A–H1A···O3B	0.85(2)	2.02(3)	2.870(5)	178(5)	*x*, *y*, *z*
		O2B–H2B···O1B	0.88(3)	1.75(3)	2.618(5)	171(7)	1-*x*, -0.5+*y*, 1-*z*
		N1B–H1B···O3A	0.84(3)	2.07(3)	2.875(5)	161(5)	*x*, *y*, *z*
L-**1**-I/D-**1**-CF_3	I	O2A–H2A···O1A	0.85(3)	1.77(4)	2.593(7)	162(9)	1-*x*, 0.5+*y*, -*z*
		N1A–H1A···O3B	0.84(3)	2.00(3)	2.836(7)	170(8)	*x*, *y*, *z*
		O2B–H2B···O1B	0.85(3)	1.78(4)	2.604(6)	163(9)	1-*x*, -0.5+*y*, 1-*z*
		N1B–H1B···O3A	0.84(3)	2.07(4)	2.896(7)	165(9)	*x*, *y*, *z*
D-**1**-*t*-Bu/L-**1**-CF_3	I	O2A–H2A···O1A	0.99(3)	1.60(3)	2.578(2)	172(3)	1-*x*, 0.5+*y*, 1-*z*
		N1A–H1A···O3B	0.84(3)	2.14(3)	2.955(3)	165(3)	*x*, *y*, *z*
		O2B–H2B···O1B	0.81(3)	1.82(4)	2.628(2)	176(3)	1-*x*, -0.5+*y*, 2-*z*
		N1B–H1B···O3A	0.82(3)	2.04(3)	2.863(3)	174(3)	*x*, *y*, *z*
